# Comparative Proteome Profile of Human Placenta from Normal and Preeclamptic Pregnancies

**DOI:** 10.1371/journal.pone.0078025

**Published:** 2013-10-18

**Authors:** Fuqiang Wang, Zhonghua Shi, Ping Wang, Wei You, Gaolin Liang

**Affiliations:** 1 CAS Key Laboratory of Soft Matter Chemistry, Department of Chemistry, University of Science and Technology of China, Hefei, Anhui, China; 2 State Key Laboratory of Reproductive Medicine, Analysis Center, Nanjing Medical University, Nanjing, Jiangsu, China; 3 Key Laboratory of Living Donor Liver Transplantation, Ministry of Public Health, Department of Liver Transplantation Center, the First Affiliated Hospital of Nanjing Medical University, Nanjing, Jiangsu, China;; Medical Faculty, Otto-von-Guericke University Magdeburg, Medical Faculty, Germany

## Abstract

To better understand the molecular mechanisms involved in pathological development of placenta in preeclampsia, we used LC-MS/MS to construct a large-scale comparative proteome profile of human placentas from normal and preeclamptic pregnancies. A total of 2636 proteins were detected in human placentas, and 171 different proteins were definitively identified between control and preeclamptic placentas. Further bioinformatics analysis indicated that these differentially expressed proteins correlate with several specific cellular processes which occur during pathological changes of preeclamptic placenta. 6 proteins were randomly selected to verify their expression patterns with Western blotting. Of which, 3 proteins’ cellular localizations were validated with immunohistochemistry. Elucidation of how protein-expression changes coordinate the pathological development would provide researchers with a better understanding of the critical biological processes of preeclampsia and potential targets for therapeutic agents to regulate placenta function, and eventually benefit the treatment of preeclampsia.

## Introduction

Preeclampsia (PE) is a multi-system disorder and a serious complication of pregnancy, which affects 5-8% of pregnancies worldwide [1,2]. It is also known as one of the leading causes of maternal or perinatal mortality and morbidity during pregnancy. Although the primary mechanism of PE is still unknown, a large amount of evidences suggest that PE could be associated with many factors such as impaired placental function, inadequate trophoblast invasion, aberrant spiral arterial remodeling, and increased apoptosis of trophoblastic cells [3,4]. Notably, recent researches indicate that impaired placental function might potentially play as an inducer during the pathological development of PE [5,6]. 

Placenta plays several critical roles in pregnancy such as preventing the rejection of the fetal allograft, transporting gases, nutrients, and waste products, and producing peptides and hormones [1-6]. Moreover, presence of the placenta is necessary for PE. Recently, some progresses have been made helping understand the molecular mechanisms of the pathological development of placenta in those patients with PE. For examples, abnormalities of trophoblast invasion and villous vascular development will lead to a failure of establishing adequate uteroplacental blood flow and may promote an exaggerated state of oxidative stress in placenta [7,8]. Dysfunctions of several molecules such as ECM antigens, focal adhesion kinase, TGF-β3, VEGF and VEGF receptors, IGF-binding protein-1, and HGF might correlate to the abnormal trophoblast invasion and vascular development, and oxidative stress in the placenta of PE [9-14]. In PE, there is excessive fibrin deposition in the placenta (e.g., overexpression of PAI-1 and PAI-2). HSP70, TNF-α, very low-density or low-density lipoprotein receptors are expressed in a spatial manner in normal placenta but their expressions change during PE development [15-18]. The aberrant up-regulations of OPG, KiSS-1, VCAM, or PDGF-AA expression in placentas of preeclamptic pregnancies might closely correlate with the pathogenesis of PE [19-22]. Several enzymes, such as villous trophoblast dypeptidyl peptidase IV, 11-β hydroxysteroid dehydrogenase, and mitogen-activated protein kinase, are also abnormally activated in PE [23-25]. Nevertheless, up to date, there have been no attempts reported to screen the regulatory factors involved in placenta of preeclamptic pregnancies by large-scale proteomic analysis. Therefore, in this study, we aim to establish a comparative proteome profile of human placentas in normal and preeclamptic pregnancies using LC-MS/MS. A total of 2636 proteins were detected in human placentas, and 171 differential proteins were identified between normal and PE pregnancies. Further functional analysis of this protein profile will provide people with deeper insights into some molecular and cellular processes during the pathological changes of placenta.

## Materials and Methods

### Sample Preparation

Placenta tissues were taken from twenty PE pregnant women and twenty healthy pregnant women according to the standard operating procedure. All of the mothers had cesarean section delivery in Maternal and Child Health Hospital of Nanjing and were within same age range and gestational weeks. All the mothers provided written informed consent. This study was approved by the Ethics Committee of Nanjing Medical University with an Institutional Review Board (IRB) number of 2012-NFLZ-32. PE was defined as a systolic blood pressure of (or above) 150 mmHg or diastolic blood pressure of (or above) 110 mmHg on two occasions in six hours. The detailed patient characteristics are presented in Table S1. For each placenta sample, 0.5 g of tissue was dissected from the maternal side of the placentas (in the central part, exclusive of calcified area) and rinsed in 0.9% saline, then frozen in liquid nitrogen prior to use.

### Protein Digestion and Dimethyl Labeling

1 mg of placenta protein from normal or PE was reduced with 10 mM DTT at 60 °C for 1 h, and then alkylated with 55 mM IAA at 37 °C for 40 min. Tryptic peptides were desalted, dried in vacuo (Speed Vac, Eppendorf), and resuspended in 100 μL of triethylammonium bicarbonate (100 mM). Subsequently, formaldehyde-H2 (573 μmol) was added into the suspension and vortexed for 2 min followed by the addition of freshly prepared sodium cyanoborohydride (278 μmol). The mixture was then vortexed for another 60 min at room temperature. After that, a total of 60 μL ammonia (25%) was added to consume the excess formaldehyde. Finally, 50 μL of formic acid (100%) was added to acidify the solution. For heavy labeling, ^13^C-D_2_-formaldehyde (573 μmol) and freshly prepared cyanoborodeuteride (278 μmol) were used. The light and heavy dimethyl-labeled samples were mixed at 1:1 ratio based on total peptide amount, which was determined by running an aliquot of the labeled samples on a regular LC-MS/MS and comparing the total signal intensities of all peptides as described [26-28]. 

### Mass Spectrometry Data Acquisition and Identification

The labeled peptides were analyzed on the LTQ-Orbitrap instrument (Thermo Fisher, USA) connecting to a Nano ACQUITY UPLC system via a nanospray source. LC-MS/MS was operated in positive ion model as described [26,27]. The analytical condition was set at a linear gradient from 0 to 60% of buffer B (CH3CN) in 150 min, and flow rate of 200 nL/min. For analysis of proteins from human placenta, one full MS scan was followed by five MS/MS scans on those five highest peaks respectively. The MS/MS spectra acquired from precursor ions were submitted to Maxquant (version 1.2.2.5) using the following search parameters: the database for search was Uniprot proteome (version20120418); the enzyme was trypsin (full cleavage); dimethylation labeling for quantiﬁcation; the dynamic modifications were set for oxidized Met (+16); carbamidomethylation of cysteine was set as static modification; MS/MS tolerance was set at 10 ppm; the minimum peptide length was 6; the false detection rates for both peptides and proteins were all set below 0.01. 

### Western Blot and Immunohistochemical Analyses

Western blot analysis was performed as described [29]. Lysates from the placentas of normal or preeclamptic pregnancy were separated on 15% SDS-PAGE gels and then the proteins were transferred to nitrocellulose membranes (Amersham Biosciences, RPR303D). The membranes were blocked in TBST containing 5% nonfat milk powder for 1 hour, incubated overnight with primary antibodies against ENG (Abcam ab70993, Cambridge, UK; 1:1000), ANXA5 (Abcam Ab14196, Cambridge, UK; 1:1000), CP (Abcam Ab48614, Cambridge, UK; 1:1000), HAB1 (Abcam Ab55081, Cambridge, UK; 1:1000), F2 (Abcam ab9262, Cambridge, UK; 1:1000), HP (Abcam Ab135835, Cambridge, UK; 1:1000), and GAPDH (Abcam Ab9485, Cambridge, UK; 1:2000), then washed three times (10 minutes each) with TBST. Then the membranes were incubated for 1 hour with alkaline phosphatase (AP)-conjugated anti-mouse or rabbit IgG (Promega, S372B, WI, USA; 1:1000). T protein levels were evaluated by the detection of activity of alkaline phosphatase using a Lumi-Phos kit (Pierce Biotechnol-ogy, KJ1243353).

For immunolocalization of ANXA5 (Abcam Ab14196, Cambridge, UK; 1:200), and CP (Abcam Ab48614, Cambridge, UK; 1:200), placenta sections (5 μm thickness) were obtained as described [29] and then fixed with 4% (w/v) paraformaldehyde (or 10% (w/v) formaldehyde) in PBS for 10 min. Then the slides were washed with PBS, blocked in 5% BSA solution for 30 min at 37 °C and incubated with primary antibodies overnight at 4 °C. Excess antibodies were removed by incubation of the slides with 0.1% Tween-20 in TBS for 15 min. Then the sections were incubated with biotinylated and streptomycin-labeled goat anti-mouse or rabbit antibody (Maixin Bio, KIT-5010, Fujian, China) for 15 minutes at room temperature. After 3 washes with TBST, the expression of the proteins in placenta sections was detected by the reaction of the second antibody with peroxidase and 3,3,9 -diaminobenzidine etrahydrochloride (DAB), and then analyzed under an Olympus BX61 fluorescence microscope.

### Statistical Analysis

For Dimethyl Labeling, the following criteria were required to consider a protein for further statistical analysis: two or more high-confidence unique peptides had to be identified, the *p* value had to be < 0.1 and the fold difference had to be greater than 1.5 or less than 0.5. Then Student’s T-test was used to find the significantly changed proteins with the SPSS software (version 18.0).

The visualized bands of western blot were quantified with Bio-Rad QUANTITY ONE software. The volumes of target bands were normalized to GAPDH. The average absolute intensity and the standard deviation were determined. The protein ratio was determined using these averaged values. Student’s T-test was used to generate *p* values. Significant difference was recognized as a *p* value less than 0.05.

### Bioinformatics Analysis

To further investigate the significance of the differentially expressed proteins, we used Pathway Studio (v5.00) software (Ariadne Genomics,MD, USA) to search their relevant molecular functions and cellular processes involved during the pathological changes of placenta. Moreover, Kyoto Encyclopedia of Genes and Genomes (KEGG) pathway and gene ontology (GO) analyses were also applied to these differentially expressed proteins as described [30].

## Results

### Global Profiling of Proteins in Human Placental Tissues

**Figure 1:**
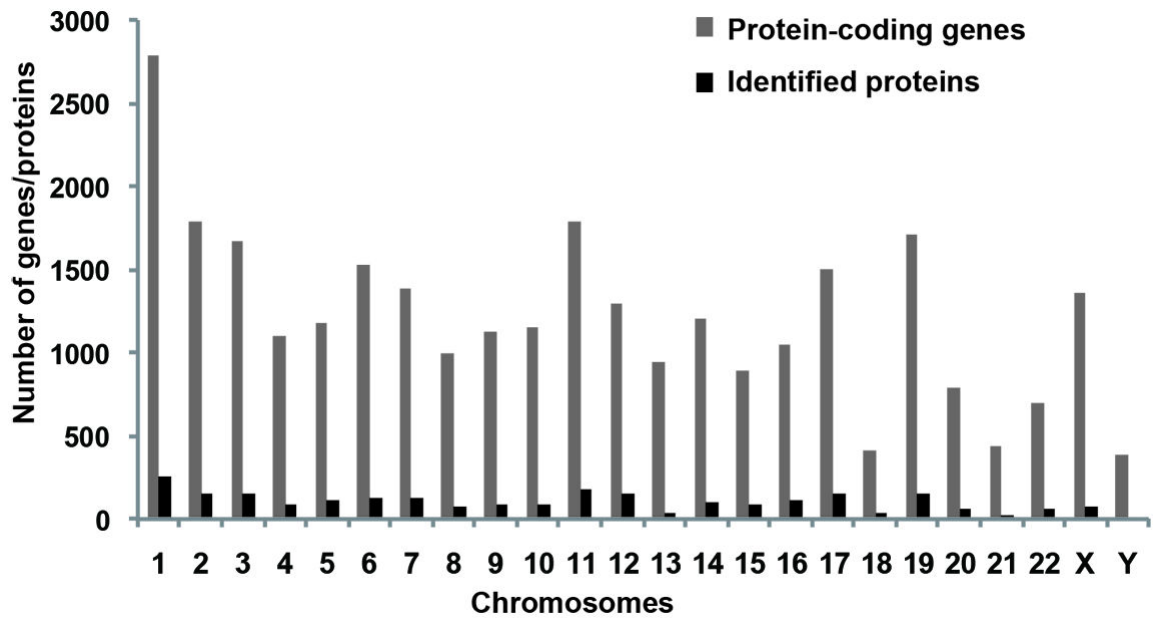
Comparison of the distributions of protein-coding genes in chromosomes with those of proteins from forty placentas (20 normal and 20 PE) identified with LC-MS/MS in this work.

**Figure 2:**
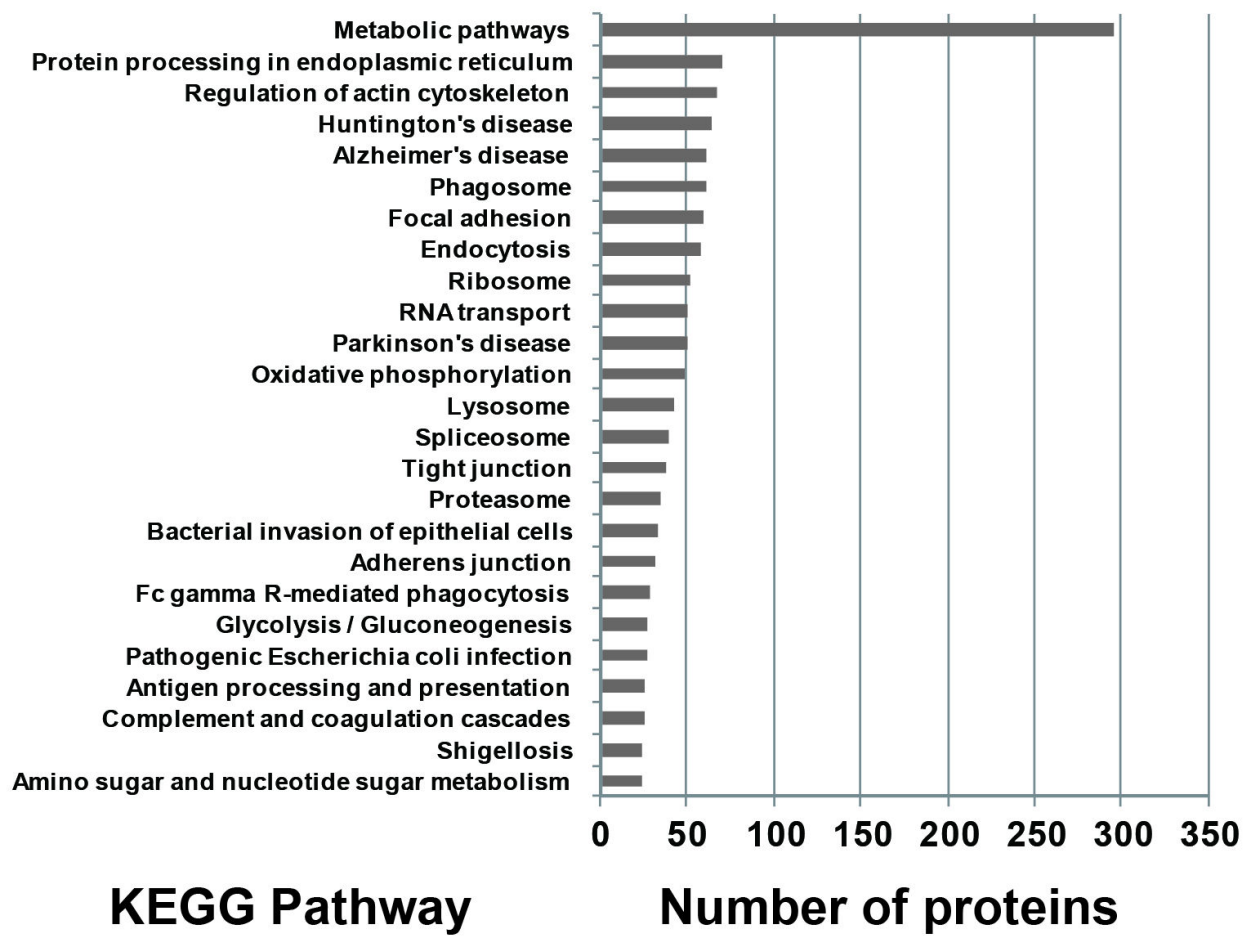
Representative significant biological pathways in which detected placental proteins are predicted to be involved. KEGG pathway analysis was performed using the identified placental proteins to evaluate which pathways are significantly represented (p < 0.05 and counts of the linked proteins > 25).

Several proteomic studies on human placenta have been reported [33-48]. However, large-scale quantitative proteomic analysis has not been reported. Therefore in this work, we used high accuracy LC-MS/MS to quantitatively detect and map proteins in the placenta at large scale. Using large-scale proteomic analysis, we identified 2636 unique proteins expressed in the human placental tissue with high confidence (two or more unique peptides with an FDR less than 1%). The distributions of these identified proteins in different chromosomes were analyzed and compared to those of protein-coding genes in human chromosomes. As shown in Figure 1, the distributions of the identified proteins in chromosomes are similar to those of human protein-coding genes. KEGG pathway analyses were performed on the identified proteins to find the important and representative pathways in human placenta that they are involved in. As shown in Figure 2, twenty-five pathways were identified for these proteins (P < 0.05 and counts of the linked proteins > 25). Most of these identified pathways are well consistent with previous observations of their roles for the development of placental during pregnancy [1-6]. For example, 297 proteins are predicted to be involved in the metabolic pathways (Figure 2). Since placenta is the critical channel between the mother and fetus for the transportation of oxygen and nutrients, the largest proportion of proteins (i.e., 297) linked to metabolic pathway could be well anticipated.

### Identification of Proteins Related to Pathological Development of Placenta in Patients with PE

To identify those differentially expressed proteins between the placenta samples from normal or preeclamptic pregnancies, we analyzed the expression patterns of 2636 proteins identified. Examination of the mass spectrometry data with Maxquant (version 1.2.2.5) revealed that 243 peptides were significantly (p < 0.05) and differentially expressed between normal and preeclamptic placentas. 171 differentially expressed proteins corresponding to these 243 protein peptides were successfully identified with LTQ-Orbitarp-Velos (Table S2). 

### Bioinformatics Analysis of Differentially Expressed Proteins

**Figure 3:**
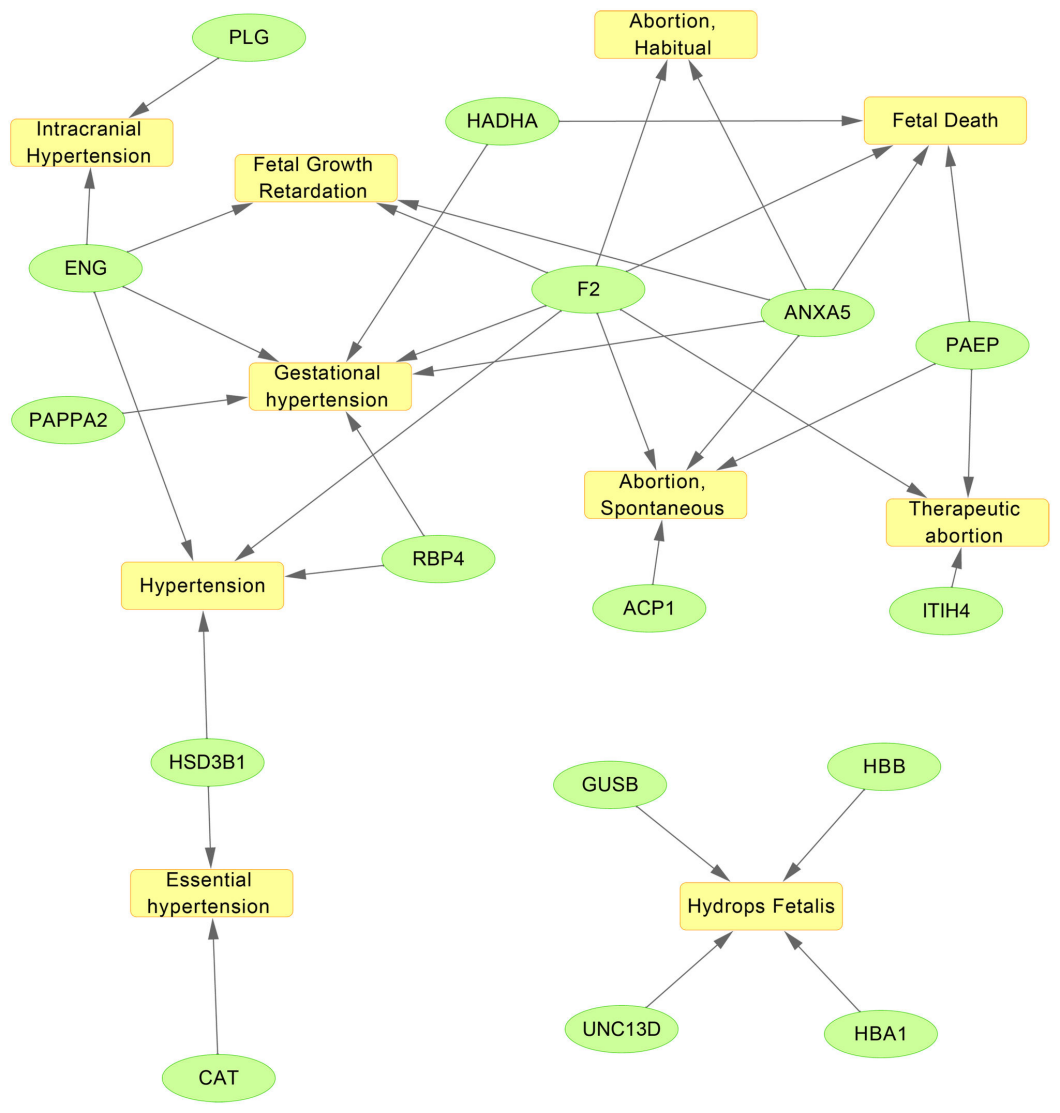
Pathways significantly represented (p < 0.05, squares) and their relevant proteins (ovals) identified as differentially expressed proteins in this work.

After further characterization the specific and unique expression patterns of the 171 proteins, we subsequently grouped these proteins into two clusters according to their expression patterns (increased expression pattern and decreased expression pattern, Table S2). Among them, 147 proteins have increased expression patterns while 24 proteins have decreased expression patterns. The identified 171 proteins were then subjected to GO analysis for further identification of important biological processes that they were significantly involved in. Indeed we found these biological processes are all present in PE development (Table S3). We ranked these processes with *p*-vaules and found that the most significant biological processes include oxygen transport, regulation of cell death, aminoglycan metabolic process, and homeostatic process (Table S3). To further analyze the networks between these important cellular processes and the 171 differentially expressed proteins, we used Pathway StudioTM for pathway analysis. A pathway map can be drawn for better visualization and understanding. Pathways significantly represented (p < 0.05, squares) and their relevant proteins (ovals) identified as differentially expressed proteins in this work are shown in Figure 3. The results indicate that these proteins are involved in pathways of "Abortion Habitual", "Intracranial HADHA Hypertension", "Fetal Growth Retardation", "Fetal Death", "Gestational hypertension", "Therapeutic abortion", etc (Figure 3).

### Western Blotting and Immunohistochemical Analyses

**Figure 4:**
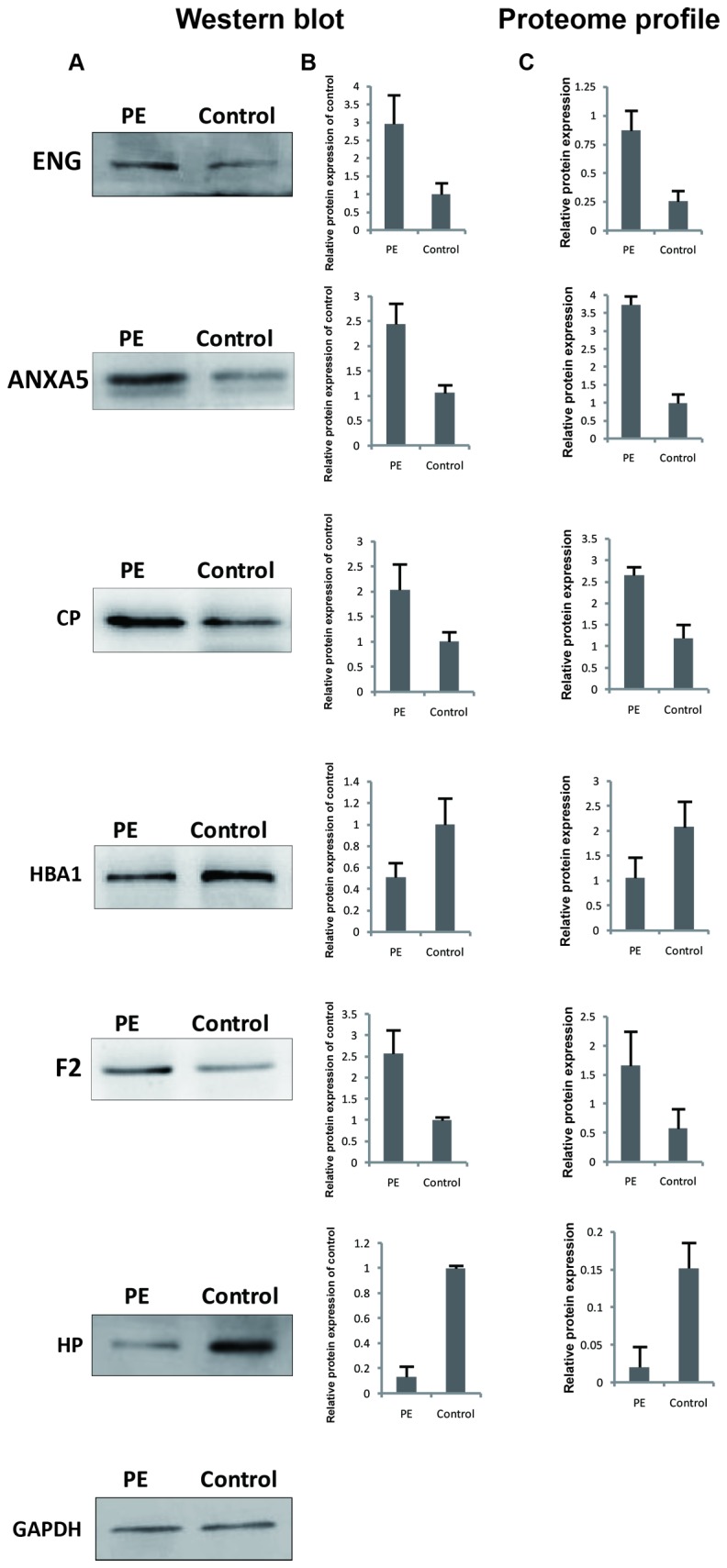
Western blotting analyses and proteome profiles of six proteins from preeclamptic and normal placentas. (A) Western blotting analyses of the six proteins. (B) Corresponding contrast analyses of the blots in A. (C) Proteome profiles of the six proteins in total protein extracts with LC-MS/MS analyses. Student’s T-test was used to generate *p* values. Significant difference was recognized as a *p* value less than 0.05.

**Figure 5:**
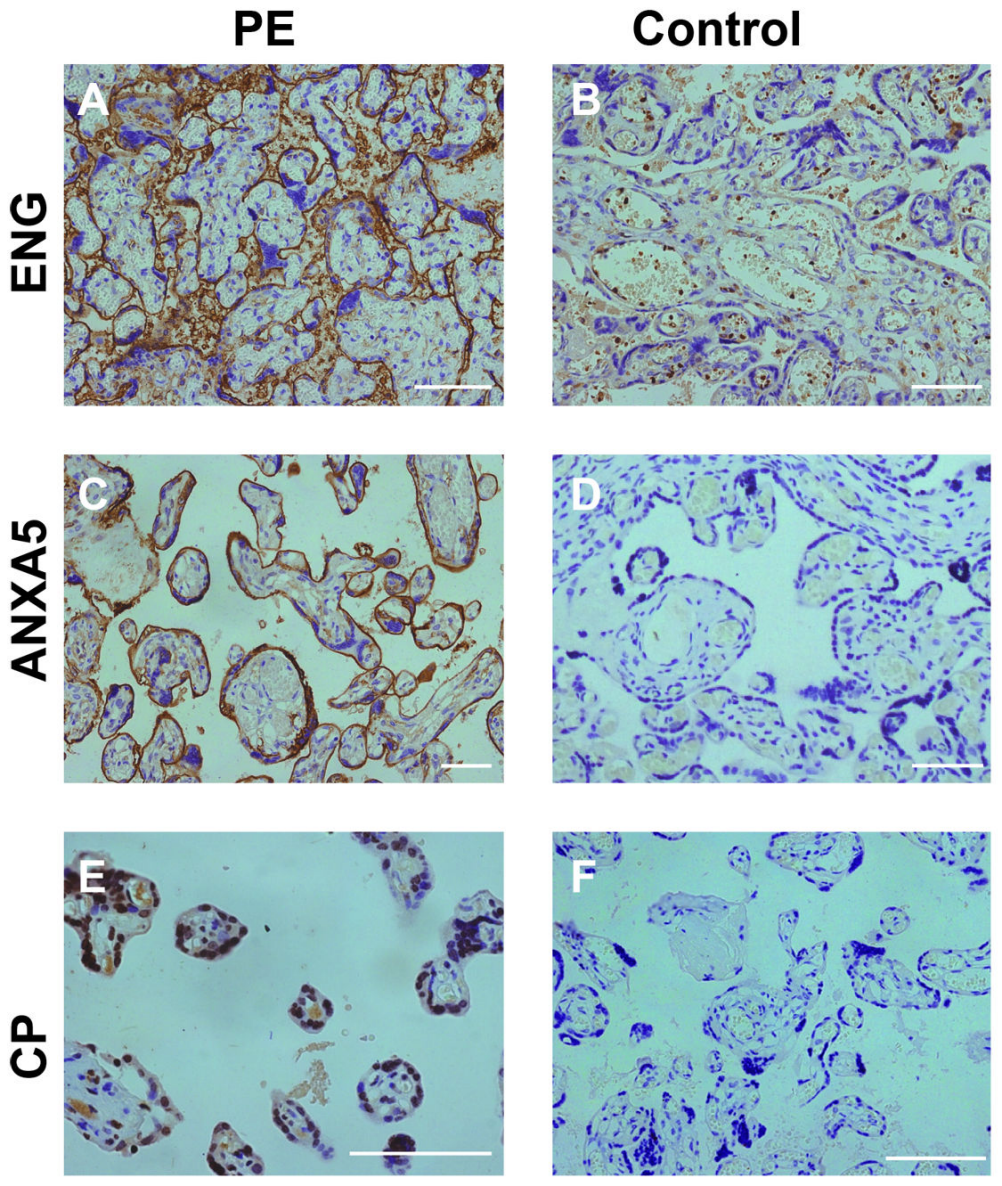
Immunolocalization of ENG (A and B), ANXA5 (C and D), and CP (E and F) in the preeclamptic and control placenta. ENG protein was localized in the syncytiotrophoblast layer (A and B). The main localization of ANXA5 was at the apical surface of placental syncytiotrophoblast (C and D). CP was localized in villi of the placenta (E and F). Scale bar: 50 μm.

We randomly selected the following six proteins (ENG, ANXA5, F2, and CP form those increased expressing proteins in PE; HBA1 and HP form the decreased expressing proteins in PE) to validate the LC-MS/MS results of the identified proteins with western blotting using GAPDH as an internal reference. As shown in Figure 4, western blotting results were essentially in agreement with those of LC-MS/MS analyses. Next, we employed immunohistochemistry to further verify the differential expression of these proteins (ENG, ANXA5, and CP) in PE or control placentas and the results satisfactorily confirmed the increased expressions of these three proteins in PE placentas (Figure 5).

## Discussion

PE is a transient disorder which develops during the last trimester of pregnancy or immediately after delivery and affects 3-8% of all pregnant women [1-3]. In pregnancies complicated by PE, an increase in mortality and morbidity of both mother and fetus has been described [1-4]. Fetal development is closely related to adequate placental growth and function. In spite of the major role of placenta in pregnancy, information about the placental proteome and its changes during PE is limited. Based on the above, we seek to obtain a comprehensive map of the differentially expressed placental proteins from PE placentas.

In this work, we performed comparative proteome studies to determine the proteins differentially expressed in human placenta between normal and preeclamptic pregnancies. 2636 unique proteins expressed in the human placental tissue were identified with high confidence. 243 protein peptides, corresponding to 171 differentially expressed proteins identified, of which 147 proteins were down-regulated while the remaining 24 proteins were up-regulated in placentas of PE. The protein lists are shown in Table S2. As far as we know, this work produced the highest number of differentially expressed proteins identified from PE placentas. 

In the list of differentially expressed proteins (Table S2), several proteins play key roles in the incidence and development of PE has been reported, such as ENG, CP, SOD, TGF-β and so on. The expressions of these proteins in our proteome profile are also consistent with those previous reports. ENG, also called CD105, is a 180-kDa homodimeric transmembrane glycoprotein expressed mainly in endothelial cells and also in many other cell types [31]. ENG is an intriguing protein that functions as an auxiliary receptor for several of the TGF-beta superfamily members. Plasma levels of soluble ENG seem to be promising as an accurate marker for PE, thus allowing early diagnosis and preventive therapy [31,32]. CP was first described as a member of a copper-containing oxidase family of enzymes. The increased levels of placental CP in PE may result in enhanced ferro-oxidative activity in this tissue, thereby oxidizing excess ferrous iron to the less toxic ferric form [49]. The syncytial CP induced by severe PE, is important in an endogenous cellular program to mitigate the damaging effects of subsequent reperfusion injury [49]. SOD is a primary antioxidant enzyme whose expression is essential for life in oxygen. In fact, lack of placentation or reduced fertility was reported in SOD-1-deficient female mice [50]. SOD is associated with oxidative stress which has gained credence as a unifying hypothesis that may explain the maternal vascular disease and placental dysfunction [45,46]. Increased urinary excretion of isoprostane or decreased antioxidant production is consistent with oxidative stress, and it precedes clinical recognition of PE [47]. In several reports, using antioxidant (e.g., vitamins C or E) to treat pregnancies with high risk of PE has showed positive effects of reducing the incidence of maternal disease [45-47]. Therefore placental oxidative stress caused by decreased SOD is likely to play a pivotal role in PE. As the fetus grows, insulin resistance becomes more apparent, causing many changes in the expression of cytokines, such as transforming growth factor beta (TGF-β) and interleukin. The differential expression level of TGF- β relating to preeclampsia patients was reported in previous research [51]. 

Many integrated bioinfomatics tools have been used to uncover the hidden biological significance with convenient functional annotation. To interpret the data easier and more efficient, we used GO analysis to analyze genes from their localizations to functions. But the pathway analysis (KEGG) was used to analyze from gene function to biological function. Detailedly, pathway analysis focuses on the regulated relationship of a group of genes in a defined biological process. This process is different from GO analysis, since GO analysis simply describes how many genes are involved. The top three networks involving the differentially expressed proteins in our study were networks of metabolic process, immune system process, and cell differentiation. The expression levels of 78 metabolism proteins were found changed. Hydroxyacyl-coenzyme A dehydrogenase and mitochondrial respiratory chain complexes were found down regulated in this work. Hydroxyacyl-coenzyme A dehydrogenase plays an essential role in the mitochondrial beta-oxidation of short chain fatty acids. This protein is associated with 3-alpha-hydroxyacyl-CoA dehydrogenase deficiency which is a metabolic disorder with various clinical presentations [52]. Mitochondria provide the main energy source for eukaryotic cells, oxidizing sugars and fats to generate ATP via oxidative phosphorylation (OXPHOS), which is accomplished by the respiratory chain. The proteins of respiratory chain such as V-type proton ATPase subunit, cytochrome b-c1 complex and NADH dehydrogenase were found down-regulated. Therefore the disruption of metabolic pathways in placenta may particular relevance to the incidence and development of PE [48]. In addition, we also firstly identified proteins such as UDP-glucose 6-dehydrogenase and prenylcysteine oxidase 1 proteins down regulated in PE placentas.

Recent data have demonstrated that down-regulated immunoregulatory system may play a key role in the development of preeclampsia [53]. In this regard, we identified 21 immunoregulatory proteins down expressed in PE patients, including interleukin-27 subunit beta, hemoglobin subunit zeta, etc. Interleukin and its receptor complex participate in a number of critical biological activities through several signaling pathways. Placental dysfunction and increased inflammation are believed to underlie the pathogenesis of severe preeclampsia (PE), which may involve interleukin-induced signaling [54]. In addition, researches have shown that maternal hemoglobin concentrations are significantly changed prior to delivery in women with preeclampsia [55]. In this work, hemoglobin subunit zeta was found down regulated about 4.9 fold. Therefore, systematical immunoactivation may be one cause of PE.

We also found that 53 proteins participating in cell differentiation and apoptosis regulation pathways were differentially expressed in PE. During human placental development, trophoblast cells differentiate through two major pathways (i.e., villous pathway and extravillous pathway). In the villous pathway, cytotrophoblast cells fuse to form multinucleated syncytiotrophoblast. In the extravillous pathway, cytotrophoblast cells acquire an invasive phenotype and differentiate into either (1) interstitial extravillous trophoblasts, which invade the decidua and a portion of the myometrium, or (2) endovascular extravillous trophoblasts, which remodel the maternal vasculature. Abnormal differentiation events, particularly the limited invasion of trophoblast cells into the uterus and the subsequent failure of the remodeling of maternal spiral arteries, are believed to induce preeclampsia [56].  Programmed cell death occurring in the extravillous trophoblast of PE is also associated to abnormal invasion [42,43]. The decreased invasion of trophoblast is associated with the existence of excessive macrophages around these arteries, secretion of TNF-α, or depletion of tryptophan [43,44]. 

In addition, Dimethyl Labeling quantitative proteomic analysis, which analyzes complex peptide mixtures with LC followed by MS/MS, might avoid many of the intrinsic shortcomings of protein-centric proteomic screens (2-D gel), particularly with respect to low-abundance molecules, due to its higher dynamic range [57]. In this work, some low-abundance proteins, such as intermediate conductance calcium-activated potassium channel protein, protein S100-A11, etc were firstly found differentially expressed in PE placentas. Further functional analyses of these PE-associating proteins are underway.

In summary, through comparative proteome analysis of placenta of normal and preeclamptic pregnancies, we constructed a protein expression profile, outlined several proteins which have been reported play key roles in the incidence and development of PE, such as ENG, CP, SOD, TGF-β, etc. We also identified proteins such as UDP-glucose 6-dehydrogenase, prenylcysteine oxidase 1 proteins, intermediate conductance calcium-activated potassium channel protein, protein S100-A11 were differentially expressed in PE placentas for the first time, to the best of our knowledge. This research might facilitate further researches on the discovery of potential biomarkers and therapeutic targets. 

## Supporting Information

Table S1 **Clinical characteristics of the patients included in this study.**
(DOC)

Table S2 **Quantitative analyses of differentially expressed proteins with LC-MS/MS.**
(XLS)

Table S3 **GO analysis for the differentially expressed proteins.**
(XLS)
